# HuB/C/D, nPTB, REST4, and miR-124 regulators of neuronal cell identity are also utilized in the lens

**Published:** 2010-11-04

**Authors:** Claudine L. Bitel, Nora I. Perrone-Bizzozero, Peter H. Frederikse

**Affiliations:** 1 Department of Pharmacology and Physiology & Rutgers-UMDNJ Integrative Neurosciences Program, UMDNJ New Jersey Medical School, Newark, NJ; 2 Department of Neurosciences, University of New Mexico School of Medicine, Albuquerque, NM

## Abstract

**Purpose:**

An interlocking network of transcription factors, RNA binding proteins, and miRNAs globally regulates gene expression and alternative splicing throughout development, and ensures the coordinated mutually exclusive expression of non-neural and neuronal forms of these factors during neurogenesis. Striking similarities between lens fiber cell and neuron cell morphology led us to determine if these factors are also used in the lens. HuR and polypyrimidine tract binding protein (PTB) have been described as ‘global regulators’ of RNA alternative splicing, stability, and translation in non-neuronal (including ectodermal) tissues examined to date in diverse species, and REST/NRSF (RE-1 Silencing Transcription Factor/Neuron Restrictive Silencing Factor) represses >2,000 neuronal genes in all non-neuronal tissues examined to date, but has not included the lens. During neurogenesis these factors are replaced by what has been considered neuron-specific HuB/C/D, nPTB, and alternatively spliced REST (REST4), which work with miR-124 to activate this battery of genes, comprehensively reprogram neuronal alternative splicing, and maintain their exclusive expression in post-mitotic neurons.

**Methods:**

Immunoprecipitation, western blot, immunofluorescence, and immunohistochemistry were used to determine the expression and distribution of proteins in mouse and rat lenses. Mobility shift assays were used to examine lenses for REST/NRSF DNA binding activity, and RT–PCR, DNA sequencing, and northern blots were used to identify RNA expression and alternative splicing events in lenses from mouse, rat, and goldfish (*N. crassa*).

**Results:**

We demonstrated that REST, HuR, and PTB proteins are expressed predominantly in epithelial cells in mouse and rat lenses, and showed these factors are also replaced by the predominant expression of REST4, HuB/C/D and nPTB in post-mitotic fiber cells, together with miR-124 expression in vertebrate lenses. REST-regulated gene products were found to be restricted to fiber cells where REST is decreased. These findings predicted nPTB- and HuB/C/D-dependent splicing reactions can also occur in lenses, and we showed Neuronal C-src and Type 1 Neurofibromatosis 1 splicing as well as calcitonin gene related peptide (CGRP) and neural cell adhesion molecule (NCAM-180) alternative transcripts in lenses. Transgenic mice with increased HuD in lens also showed increased growth associated protein 43 (GAP43) and Ca^++^/Calmodulin dependent kinase IIα (CamKIIα) HuD target gene expression in the lens, similar to brain.

**Conclusions:**

The present study provides the first evidence this fundamental set of regulatory factors, previously considered to have a unique role in governing neurogenesis are also used in the lens, and raises questions about the origins of these developmental factors and mechanisms in lens and neuronal cells that also have a basic role in determining the neuronal phenotype.

## Introduction

**Figure 1:**
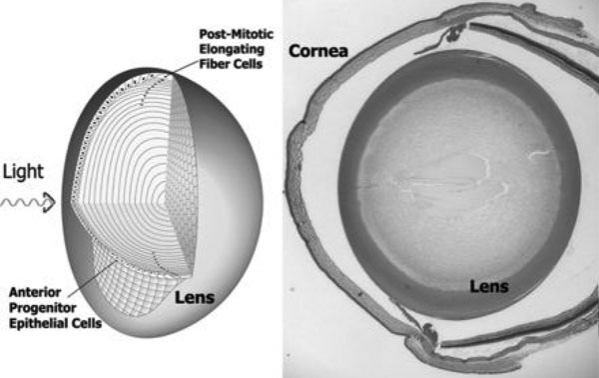
Structure of the eye and cellular lens; organization of vertebrate lenses. Small cuboidal epithelial cells cover the anterior surface. At the anterior/posterior equator, these cells exit the cell cycle and begin to elongate as they move into the interior. A few hundred microns into the lens, fiber cells undergo a final stage of terminal differentiation where they lose cell nuclei and organelles. At right is a histological section of an adult mouse eye.

Neurons are among the most ancient of all specialized cells in the body, and are distinguished by their elongated cellular processes and unique vesicle transport system [1]. Neural development utilizes sets of molecular factors that act as genetic switches and form an interlocking network that regulates the expression of neuronal genes, alternative splicing and translation, and suppresses >2,000 genes in non-neuronal cells throughout development [2-5]. A hallmark of this network is the coordinated mutually exclusive expression of non-neural versions of these factors and their neuronal counterparts in neural progenitor cells and post-mitotic neurons respectively. It is estimated >90% of all genes undergo alternative splicing, and a large percentage occurs in neurons [2,6-8]. Over 50 years ago, electron microscopists began noting key features of post-mitotic lens fiber cells and neurons that are remarkably similar. Lens fiber cells are ~5 µm in diameter and also undergo a process of pronounced cell elongation, becoming >1 cm in length in some animals [9-11]. In vertebrates, progenitor epithelial cells cover the lens anterior surface (Figure 1). At the anterior/posterior equator these cells exit the cell cycle and begin to elongate, directed by factors in the anterior aqueous and posterior vitreous chambers [12]. As this process begins, Byers and Porter [10] noted in the 1960s that microtubules oriented in the direction of cell elongation appear, which have a ‘Ferris-wheel’ construction when viewed end-on that is consistent with polarized vesicle transport [11]. Fiber cell lateral surfaces are lined with ball-like protrusions that are enriched with F-actin and exclude microtubules and are coated with Clathrin/AP-2 complexes, which led to detailed comparisons with dendritic spines [11,13,14]. These basic similarities in cell morphology suggest related cell biology processes, gene expression, and associated regulatory factors may also be involved in lens biology.

An important regulatory factor that suppresses neuronal gene expression in non-neuronal cells and neural progenitor cells throughout development is REST/NRSF (RE1-silencing transcription factor; Neuron Restrictive Silencing Factor). REST binds Neuron Restrictive Silencer Elements (NRSEs) in >2,000 neuronal genes and represses their expression [15-17], and was termed a ‘master regulator of the neuronal phenotype’ [18]. Consistent with its role as a potent repressor of large numbers of neuronal genes in all non-neural tissues examined to date, REST is also required for ectodermal tissue development in vertebrates. Conversely, REST suppression ectopically activated these genes in non-neuronal tissues and induced neuronal differentiation. Likewise, mutated REST:NRSE DNA binding sites produced ectopic expression of a large number of target genes in non-neuronal tissues, including ectodermally derived tissues examined to date [5,19,20], but not the lens. As REST is down-regulated neural progenitors exit the cell cycle, allowing these neuronal genes to become activated [3,5,21]. Agreeing with these findings, maintaining REST expression prevents neurogenesis. REST is also necessary for stem cell pluripotency, and figures prominently in tumor suppression [22,23]. An alternatively spliced isoform, REST4, was found to be produced exclusively in post-mitotic neurons, and lacks a key repressor domain [16,24]. REST4 is thought to facilitate silencing of REST repression; however, these mechanisms are not yet clear. REST4 was found to bind NRSEs in vitro, but with >20-fold lower affinity. In vitro, REST4 was also shown to interact with REST, and might also provide a mechanism to sequester REST and further relieve REST gene suppression [25].

Genetic switches in this global regulatory network that govern neurogenesis also operate at the post-transcriptional level. Similar to REST/REST4, RNA binding proteins (RBPs), which include Hu proteins and polypyrimidine tract binding proteins that are each encoded by separate genes, regulate alternative RNA splicing, translation, as well as mRNA half-life and localization in cells throughout development in many species examined [2,4,26-28]. HuR and polypyrimidine tract binding protein (PTB, PTBP1) have been shown to be expressed in all non-neuronal cells examined to date throughout development, and this included tissues of ectodermal origin examined in several diverse species. Neuronal HuB/C/D and neuronal PTB (nPTB, PTBP2) were characterized as having exclusive expression in post-mitotic neurons, and have fundamental roles in determining neuronal cell identity [2,7,29]. These RBPs also interact with the translation machinery to specifically increase expression of target genes. Moreover, this latter activity was shown to be required for neurite outgrowth [4,28,30]. For example, HuD is one of the earliest markers of neurogenesis, and introduction of HuD into cells initiates process formation, and is also required for neuronal development [31,32]. By contrast, HuR and PTB have been referred to as ‘non-neural’ and ‘ubiquitously expressed’ in the literature, and have essential roles in the development of non-neuronal tissues, including tissues of ectodermal origin examined in animals that include *Xenopus* and sea urchins [33-35].

Tissue-specific miRNAs also regulate neuronal gene expression, and are also incorporated into this regulatory network. Lim et al. [36] showed tissue-specific miRNAs help establish cell identity by suppressing inappropriate gene expression in a given cell type. Approximately 22 nucleotide miRNAs bind transcripts to tag them for degradation or inhibit translation. In brain, several studies had characterized miR-124 as neuron-specific and showed it suppresses hundreds of non-neuronal transcripts in post-mitotic neurons. Previously, we determined that miR-124 is also uniquely expressed in adult rat and mouse lenses [37], and subsequently others showed miR-124 is highly expressed in other eye tissues, as well as in the regenerating newt lens [38,39]. Conaco et al. [15] showed the *miR-124* gene is also a target of REST repression in non-neural cells. Thus, in post-mitotic neurons miR-124 is expressed and suppresses PTB and its non-neuronal alternative splicing activities. This in turn allows nPTB to be expressed and neuronal alternative splicing to occur [18] (diagrammed below). Conversely, REST repression of miR-124 in non-neural cells permits PTB expression that in turn suppresses nPTB and its neuronal splicing activities, and promotes PTB-dependent non-neuronal alternative splicing in non-neural cells. Together, these factors help to coordinate the differential expression and mutually exclusive alternative splicing of thousands of genes during neurogenesis, including their own.

In a previous study, we demonstrated several genes considered to be neuron-specific are also expressed during embryonic fiber cell development [40]. For example, we showed that synapsins 1, 2, and 3 were expressed predominantly along the axial length of rapidly elongating fiber cells during embryonic development. Synapsin 1 (syn1) and βIII-tubulin (tubb3) have been extensively characterized as REST/NRSF targets of repression in non-neuronal cells. Syn1 neuronal specificity was also shown in an array of tissues (except lens) in sensitive radioactive promoter/reporter gene assays in transgenic mice [41]. Here, we began an analysis of the mutually exclusive expression of these regulatory factors in lens progenitor and post-mitotic fiber cells. We found that syn1 and tubb3 are also predominantly expressed in adult post-mitotic fiber cells at the lens periphery. We showed PTB, HuR, and REST are expressed almost exclusively in progenitor epithelial cells, and that their expression is replaced by nPTB, HuB/C/D and REST4 in post-mitotic lens fiber cells. We also demonstrated REST:NRSE DNA binding activity in lenses. When we tested lenses for alternative transcript splicing reactions characterized as neuron-specific to date, we showed nPTB- and HuB/C/D dependent reactions can also occur in lenses. For example, we found that neuronal Type 1 Nf1 and Neuronal C-src spliced products are also the major alternative transcript produced in lenses. We also demonstrated an additional key member of this regulatory network, miR-124, is expressed in fish as well as mammalian lenses. An examination of transgenic mice with increased HuD in the lens also showed predicted increased expression of HuD target genes in the lens, consistent with effects shown in the brain in this model. Together, the present findings provide evidence that these molecular switch components are also uniquely shared in the lens.

## Methods

### Immunological detection of proteins

Animals were used according to NIH guidelines and IACUC approved protocols. Immunoblots were prepared using lens and brain tissue samples from 4 week old mice (C57) and rats (Sprague-Dawley). Transgenic mice expressing HuD have been characterized and are described elsewhere [42]. Lenses were removed using established procedures (D. Garland, Univ. Penn. Philadelphia, PA, personal communication). Tissue samples in SDS sample buffer were resolved on 10% Bis-Tris gels (Invitrogen, Carlsbad, CA) and blotted to filters. Filters blocked in 5% dry milk in PBS with 0.01% Tween-20, were probed with antibodies according to the supplier. Secondary antibodies conjugated to HRP raised against species-specific immunoglobulins (Jackson Immunologicals, West Grove, PA) were used to visualize immune complexes by chemiluminescence (Amersham, Piscataway, NJ).

Immunoprecipitations used 200 µg of lens proteins in RIPA (Radio-Immunoprecipitation Assay) buffer (Sigma, St. Louis, MO) with protease inhibitors (Calbiochem, San Diego, CA) incubated with anti-REST mAb and incubated overnight at 4 °C. Immune complexes were separated with Protein G magnetic beads (Invitrogen), and washed 3× in RIPA buffer. Proteins were released using Glycine buffer, pH 2.8. Immunoprecipitated proteins were resolved on gels as above and probed with anti-REST and REST4 specific antibodies listed below.

For in situ immunofluorescence detection of proteins, whole eyes fixed in buffered 4% paraformaldehyde, were used to prepare paraffin sections. Sections de-waxed in xylenes and alcohol washes, were blocked in PBS with 10% serum. Primary antibodies were diluted as suggested by suppliers. In addition to producing distinct protein distribution patterns in the same or adjacent sections with several antibodies raised in different species, controls in which no primary antibody was added detected no signal.

Antibodies used in this study included: mAb anti-REST NH_2_-terminal (gift of D. Anderson, Cal Tech, Pasadena CA), anti-COOH-terminal REST (Millipore, Lincoln Park, NJ), rabbit anti-REST4 3121, 3122 (gift of N. Buckley, King’s College London, UK), chicken anti-neuronal βIII-tubulin (Aves, Tigard, OR), rabbit anti-neuronal βIII-tubulin (T2200; Sigma), mAb anti-neuronal βIII-tubulin (SDL.3D10; Sigma), rabbit mAb anti-C-terminal neuronal βIII-tubulin (Epitomics, Burlingame, CA), mAb anti-Synapsin 1 (BD Biosciences, San Jose, CA), mAb anti-Synapsin 1 (Millipore), mAb anti-NCAM-180 (4d; Developmental Studies Hybridoma Bank, Iowa City, IA), rabbit anti-GAP43 (Novus, Littleton, CO), rabbit anti-Synaptophysin 1 (Genscript, Piscataway, NJ), mAb anti-Synaptotagmin 1 (Calbiochem), rabbit anti-CamKIIα (Sigma), mAb anti-GAPDH (Abcam, Cambridge MA). COOH-terminal and NH_2_-terminal rabbit anti-PTB and anti-nPTB antibodies (gift of D. Black, UCLA, Los Angeles CA). Human anti-HuB/C/D was a gift of H. Lou (Case Western Reserve, Cleveland, OH; see also [27] for review), mAb anti-HuD (Santa Cruz, Santa Cruz, CA), mAb anti-HuR (Santa Cruz).

### RT–PCR analysis of transcripts

Lens and brain RNAs were purified with tri-reagent (Sigma). Reverse transcriptase reactions (Superscript; Invitrogen) used oligo-dT or random hexamer primers. The PCR primers used are listed in Appendix 1. All PCR primer pairs corresponded to different exons not contiguous in the genomic DNA, except for REST. PCR reaction products produced with Taq polymerase (Applied Biosystems, Carlsbad CA) were resolved by molecular weight on agarose gels, and isolated using purification kits (Qiagen, Valencia CA). Each PCR product was sequenced by the dideoxy method, to show that the complete amplified product sequence matched data in the NIH NCBI database, and the presence of exon junctions consistent with spliced transcript sequences is also confirmed in specific regions identified in DNA chromatograms (Applied Biosystems).

### Gel mobility shift assay of protein/DNA binding

Crude lens protein extracts were prepared by homogenizing 100 mg lens tissue in 10 mM HEPES buffer, pH 7.9, supplemented with 10 mM KCl, 0.1 mM EDTA, 0.1 mM EGTA, 1 mM dithiothreitol (DTT), 0.1 mM phenylmethylsulphonyl fluoride (PMSF) and incubated on ice [43]. ds-NRSE oligonucleotide probes were end-labeled with T4-polynucleotide kinase (Invitrogen) and γ^32^P-ATP (Amersham) as recommended by the manufacturer, and purified on G-25 mini-spin columns (Roche, Nutley NJ). Unlabelled probe and nucleotide-substituted NRSE probe were used as competitors to ascertain binding specificity as described elsewhere [16,43]. Briefly, ~50,000 cpm labeled probe was incubated with protein extract in the absence or presence of unlabelled competitor at 25- or 100-fold excess. After 30 min on ice, reactions were resolved by molecular weight on 6% TBE acrylamide gels which were dried and exposed to film.

### Northern blots

Northern blot assays of miRNA expression in total RNA samples from lens and brain, and muscle specific miRNA negative controls are described elsewhere [37]. Total RNAs from mouse, rat, or goldfish (*N. crassa* tissue gift of N. Ingoglia, UMDNJ, Newark NJ) lens and brain tissues were resolved on 14% urea-acrylamide gels (National Diagnostics, Atlanta GA) and blotted to filters (Amersham). Filters were incubated overnight in blocking buffer with carrier yeast RNA and herring DNA (Sigma). Oligonucleotides radiolabeled with polynucleotide kinase (Invitrogen) were purified on G-25 mini-spin columns (Roche) and used to probe filters in the same buffer. Filters were washed in stringent buffers, and autoradiograms were obtained.

## Results

### Neuronal gene products suppressed by REST in non-neural cells throughout the body are expressed in adult lenses predominantly in elongating fiber cells

**Figure 2:**
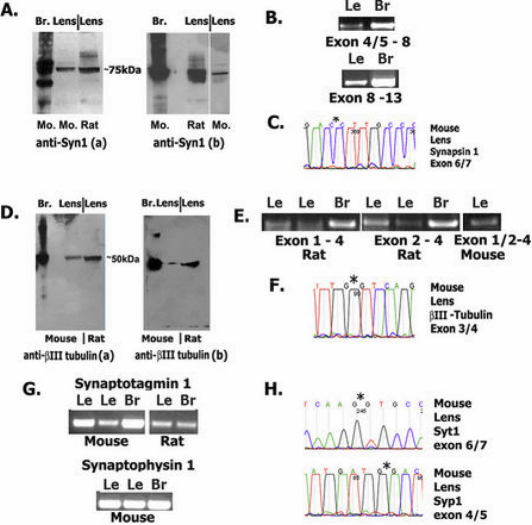
REST (NRSF) regulated neuronal genes are activated in the lens. **A**: Immunoblot detection of Synapsin 1 in lens and brain (a: Millipore; b: BD Biosciences). **B**: Amplification of Synapsin 1 transcripts from lens and brain. **C**: Representative sequence identifying exon junction in cDNA products. **D**: Immunoblot detection of neuronal βIII-tubulin in lens, and brain (a: rabbit antibody, Sigma; b: chicken antibody, Aves). **E**: Amplification of neuronal βIII-tubulin transcripts from lens and brain. **F**: Region from complete amplified product sequence identifying in frame exon junction in amplified transcripts. **G**: Amplification of Synaptotagmin 1 and Synaptophysin 1 transcripts from lens and brain. **H**: Region from amplified product sequence identifying in frame exon junctions in amplified transcripts. Asterisks indicate splice junctions.

**Figure 3:**
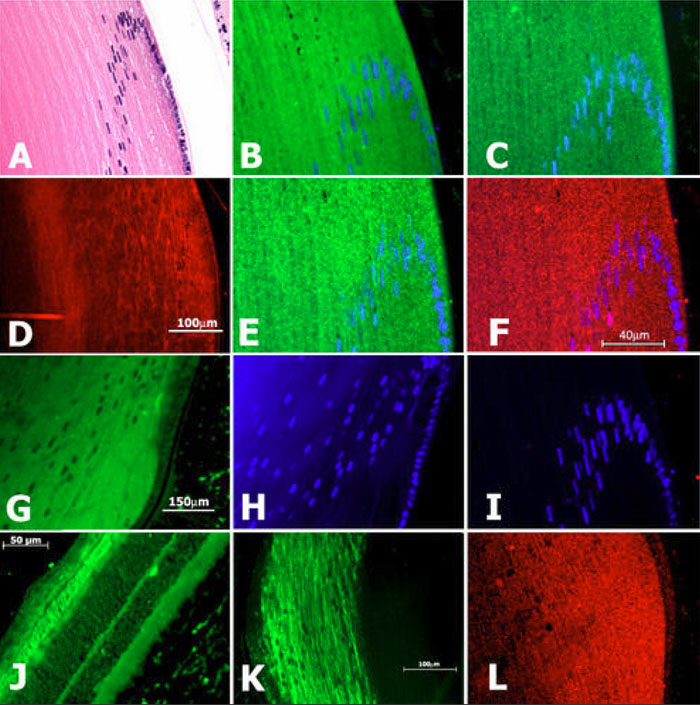
REST regulated neuronal genes are activated in post-mitotic fiber cells. A: Hematoxylin and eosin stained mouse lens. **B**: Mouse mAb βIII-tubulin. **C**: Rabbit mAb anti-βIII-tubulin. **D**: Chicken anti-βIII-tubulin. **E**: Anti-Synaptophysin 1. **F**: Mouse mAb Synaptotagmin 1. **G**: Rabbit anti-Synapsin 1. **H**: DAPI as in **G**. **I**: No primary antibody control (lo), DAPI. **J**: Synapsin 1 in retinal neuronal layers (autofluorescence is seen in photoreceptor layer). **K**: mAb βIII-tubulin. **L**: Synaptotagmin 1 in peripheral fiber cells.

Syn1 and tubb3 have been characterized extensively as examples of targets of REST repression in non-neural cells, and have been described as neuron-specific [3,44,45] before our examination in the lens [40]. To demonstrate syn1 expression in adult lenses, we probed immunoblots containing equal amounts of 14 day (d) rat and 28 days mouse proteins using two anti-syn1 antibodies that each identified syn1 in mouse and rat lens and brain (Figure 2A). We also amplified syn1 from lenses (Figure 2B), and sequenced the cDNA products to confirm they were derived from syn1 transcripts. Figure 2C shows short regions from these DNA sequences identifying in frame exon junctions not encoded in the genome. In addition, ‘no-RT’ negative controls produced no product (not shown). To examine the distribution of syn1 protein in lenses, we probed eye sections and observed substantial syn1 expression in elongating lens fiber cells at the periphery of adult lenses, and little syn1 signal in progenitor epithelial cells along the anterior lens surface (Figure 3G). Expression of syn1 strongly in fiber cells is consistent with syn1 vesicle transport functions in neurons and the appearance and distribution of polarized microtubules in elongating fiber cells [10]. Similarly, we identified tubb3 on immunoblots of total mouse and rat lens proteins using mAb and polyclonal tubb3 antibodies (Figure 2D). In addition, we confirmed *tubb3* cDNA sequences of amplified products also contained in frame exon junctions, shown in Figure 2E,F. Examination of tubb3 in lens sections with antibodies raised in different species each detected tubb3 protein predominantly in elongating lens fiber cells (Figure 3B-D,K). We observed that syn1 and tubb3 decreased significantly in fiber cells deeper into the interior, at a boundary ~200–300 µm from the lens surface in mouse and rat. This position also corresponds with the disappearance of microtubules in mammalian lenses [11]. Near this location, lens fiber cells undergo final terminal differentiation where organelles are lost in a process that has been compared with red blood cell differentiation [46,47], and corresponds with DAPI stained cell nuclei present in the plane of the histological section. In addition, we determined the lens expression of two other REST target genes in non-neuronal cells: Synaptotagmin 1 (*syt1*) and Synaptophysin 1 (*syp1*) [44,45]. We amplified *syt1* and *syp1* transcripts from lenses, again identifying splice junctions in complete cDNA product sequences (Figure 2G,H). We also showed both proteins are also substantially restricted in expression in peripheral fiber cells in adult lenses (Figure 3E,F,L). In addition, we showed *syn1* expression in retina, which has been considered part of the central nervous system (Figure 3J). By contrast, studies have shown that skin and cornea, ectodermally derived tissues that are also innervated by neuronal processes, express tubb3 protein specifically within elongated neuronal processes only [48,49]. We also note expression of these genes mainly in fiber cells of adult lenses agrees with our study of their expression during embryonic lens development.

### REST is predominantly expressed in anterior lens epithelial cells, and ‘neuron-specific’ alternatively-spliced REST4 in post-mitotic elongating fiber cells

**Figure 4:**
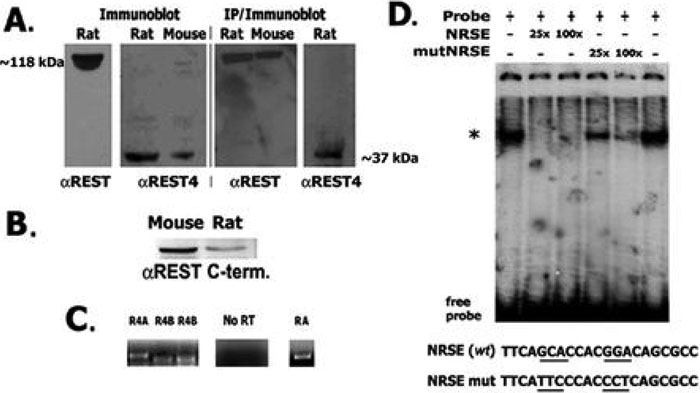
REST/NRSF, and neuron-specific alternatively-spliced REST4 are produced in lenses; identification of functional NRSE binding activity in the lens. **A**: Immunoblot and IP detection of REST and REST4 in the lens. Left panels: mAb anti-REST and anti-REST4 3121. Right panels: Immunoblot of lens proteins immunoprecipitated with NH_2_-terminal anti-REST mAb, probed with mAb anti-REST or anti-REST4 3122. **B**: Detection of REST in lenses using anti-COOH-terminus REST. **C**: Amplified REST4 (R4A, R4B) and REST (RA) transcripts from lenses. **D**: Gel mobility shift assay identifying REST:NRSE DNA binding activity in lens extracts. NRSE and nucleotide-substituted NRSE sequences are shown below. Asterisk indicates mobility shifted complexes. NRSE and nucleotide substituted competitor were added at 25- and 100-fold excess.

Our experiments showed expression of *REST* gene targets predominantly in post-mitotic fiber cells is consistent with decreased REST in these cells, and suggests further that REST4 is also produced in the lens in post-mitotic fibers. To examine these possibilities, we first determined that REST and REST4 are both expressed in the lens. mAb anti-REST and anti-REST4 specific antibodies identified both REST and REST4 on immunoblots of mouse and rat proteins from lens and brain (Figure 4A). To confirm these findings, REST and REST4 were also detected on immunoblots of lens proteins first immunoprecipitated with NH_2_-terminal-specific anti-REST (Figure 4A). Full-length REST was also identified on immunoblots of mouse and rat lens proteins using COOH-terminal specific antibodies (Figure 4B). We also amplified *REST* and alternatively spliced *REST4* transcripts from lenses by RT–PCR (Figure 4C), and confirmed the cDNA product sequences (not shown).

**Figure 5:**
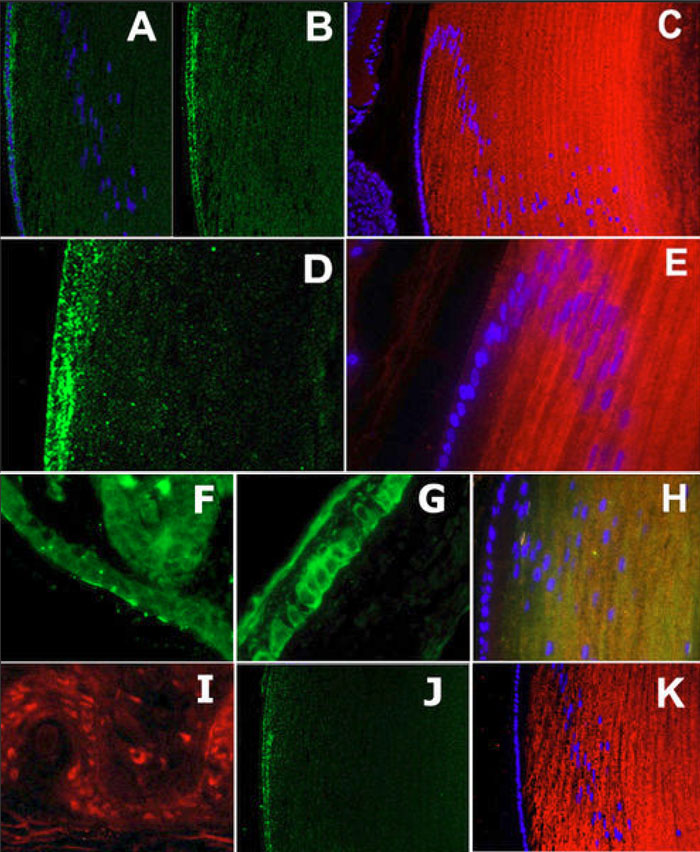
Mutually exclusive expression of REST in progenitor epithelial cells, and alternatively spliced REST4 in post-mitotic elongating lens fiber cells. **A**, **B**: Anti-C-terminal REST (100×); nuclei are stained with DAPI in panel **A**. **C**: Anti-REST4 3121 (50×). **D**: Anti-COOH-terminal REST (200×). **E**: Anti-REST4 3122 (200×). **F**: REST detected in rat skin. **G**: REST detected in Cornea. **H**: Overlapping REST4 and tubulin detection in lens fiber cells; cell nuclei are DAPI stained. **I**: HuR detected in rat skin. **J**: Anti-REST. **K**: Anti-REST4; nuclei are stained with DAPI in lens.

To determine if REST DNA binding activity is present in lenses we used standard gel mobility shift assays and labeled NRSE oligonucleotide probes and demonstrated NRSE binding activity is present in the lens (Figure 4D). Mobility shifted REST:NRSE complexes were competed by excess unlabelled “self” oligonucleotides, but not effectively with oligonucleotides having substitutions in nucleotides critical for REST binding [16]. We next examined the distribution of REST and REST4 in lenses and observed a strong mutually exclusive pattern of expression for these two isoforms (Figure 5), which is consistent with fiber cell expression of REST target genes shown above. REST decreased substantially near the lens equator and REST4 was strongly detected only in post-mitotic fiber cells in the lens interior. In addition to identifying discrete patterns detected with REST and REST4 antibodies in adjacent lens sections using the same procedures, ‘no primary antibody’ negative controls produced no signal similar to the control in Figure 3I. As predicted, REST4 and tubb3 protein overlapped substantially in lens fibers (Figure 5H). REST4 was detected throughout the cytoplasm of fiber cells and in regions distal from the cell soma and nuclei. For comparison, we also probed cornea and skin tissue sections and observed an analogous pattern of REST expression in both of these ectodermally derived tissues, where no specific nuclear localization was detected (Figure 5F,G; see also discussion below). In addition, REST4 was not detected in non-neural tissues in studies cited above. The ability to detect REST4 also stopped at the boundary where final lens fiber cell terminal differentiation begins, which again agrees with the distribution of REST-regulated gene products shown above.

### PTB (PTBP1) and neuronal nPTB (PTBP2) are expressed in lenses with preferential mutually exclusive distributions in epithelial and fiber cells

**Figure 6:**
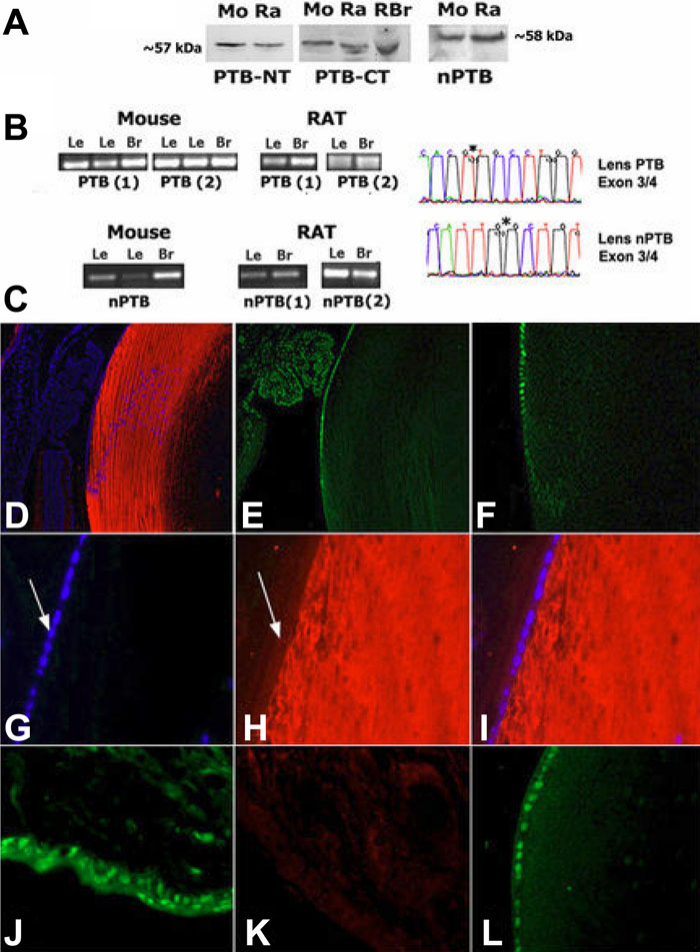
PTB (PTBP1) is expressed in progenitor epithelial cell and neuronal nPTB (PTBP2) in post-mitotic lens fiber cells. **A**: Immunoblot detection of PTB and nPTB in mouse and rat lens and brain tissue. PTB-NT: anti-NH_2_-terminus PTB, PTB-CT: anti-COOH-terminus PTB, and anti-nPTB. **B**: Left: Amplified PTB and nPTB transcripts from lens and brain. Right: Region from amplified DNA sequence product identifying in frame exon junctions in lens. **C**: Immunofluorescence detection of PTB and nPTB in the lens. **D**: anti-nPTB (100×), **E**: anti-PTB-NT (100×), **F**: anti-PTB-CT (100×), **G**: DAPI nuclear stain/no 1o control (200×), **H**: anti-nPTB (200×), **I**: overlay panel **G** and **H**. **J**: For comparison PTB is detected in cell nuclei in rat skin, **K**: anti-nPTB detects little or no protein in rat skin, **L**: Detection of PTB in cell nuclei in epithelial cells on the anterior lens surface.

Although factors involved in ‘neuron-specific’ alternative splicing of REST4 have not yet been determined, expression of alternatively spliced REST4 in the lens indicates neuronal splicing machinery may also be present in the lens. PTB and nPTB are RBPs that mediate alternative splicing during neurogenesis, and are encoded by separate genes [2]. PTB suppresses nPTB expression in neural progenitors and non-neuronal cells. In post-mitotic neural cells, miR-124 suppresses PTB [8,29] to allow neuronal alternative splicing (see diagram below) [2,26]. This PTB/nPTB system is estimated to regulate up to 25% of alternative splicing in mammals [2,7,8]. To test if PTB and nPTB are expressed in lenses we probed immunoblots of lens proteins with PTB and nPTB specific antibodies (gift of D. Black, UCLA, Los Angeles, CA), and identified both proteins in the lens (Figure 6A). We also confirmed their expression at the RNA level using RT–PCR to amplify spliced transcript regions expressed by PTB and nPTB genes (Figure 6B). When we examined PTB and nPTB proteins in situ, we again observed a predominantly mutually exclusive expression pattern of these factor isoforms in progenitor epithelial cells and post-mitotic fiber cells. We noted that PTB in anterior epithelial cells was almost exclusively detected within cell nuclei, and nPTB was observed throughout the cytoplasm of post-mitotic fiber cells, which may indicate nPTB interaction with cytoplasmic transcripts. For comparison, PTB but not nPTB was detected in rat skin, consistent with studies cited above (Figure 6C). Moreover, PTB in skin cells was also primarily within cell nuclei. We also identified protein corresponding to the NCAM-180 neuronal isoform in lens fiber cells and detected epitopes specifically encoded by the nPTB dependent spliced exon, and is discussed further below [50].

### Globally expressed HuR and neuronal HuB/C/D are expressed in the lens in mutually exclusive distribution in epithelial and fiber cells

**Figure 7:**
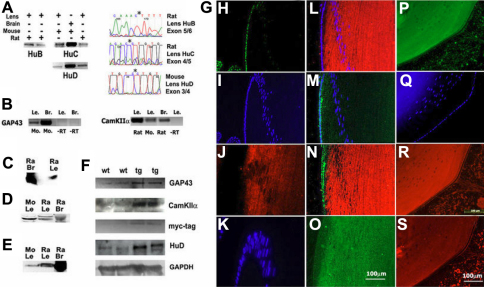
HuR is expressed in progenitor lens epithelial cells, and neuronal HuB/C/D in post-mitotic elongating fiber cells. **A**: Amplification of HuB, HuC, and HuD transcripts from lens and brain; right: Region from sequenced product showing in frame exon junctions in amplified transcripts. **B**: Amplification of the HuD target transcripts: GAP43 and CamKIIα from wt mouse and rat lens and brain. **C**: Immunoblot detection of HuR in lens and brain. **D**: Immunoblot of HuB/C/D in lens and brain (Human anti-HuB/C/D). **E**: GAP43 protein detected in wt lens and brain. **F**: Increased expression of GAP43 and CamKIIα detected on immunoblots of wt versus transgenic mouse lenses expressing myc-tagged HuD in the lens; unchanged GAPDH levels are shown for comparison. **G**: Immunofluorescence detection in rat lens: **H**: mAb anti-HuR (200×), **I**: DAPI stained nuclei as in panel **H** (100×), **J**: Human anti-HuB/C/D, **K**: DAPI stained nuclei as in panel **J** (200×), **L**: Human anti-HuB/C/D (100×), **M**: anti-COOH-terminal REST, **N**: overlay of panels **L** and **M**, **O**: Syn1 expression in post-mitotic fiber cells (BD Biosciences, 200×), **P**: GAP43 in wt lens, **Q**: DAPI stained nuclei as in panel **P**, **R**: CamKIIα in wt lens, **S**: mAb anti-HuD detection in wt mouse lens (**L**-**P**, Santa Cruz).

Hu proteins are also known as ELAV proteins in reference to the *Drosophila* homolog: Embryonic Lethal Affecting Vision, which is required for neuronal development in flies. Here, we examined lenses for expression of this additional class of RBPs which has also been shown to have a fundamental role in neural and non-neural alternative splicing, and in establishing neuron cell identity in vertebrates. To determine if genes previously characterized as “neuron-specific” that encode HuB, HuC, and HuD proteins are also expressed in the lens we amplified their transcripts from rat and mouse lenses (Figure 7A). Human anti-HuB/C/D and anti-HuR antibodies were used to detect these proteins on immunoblots (Figure 7C,D). Human antibodies used in these experiments specifically detect neuron-specific Hu proteins and have been extensively characterized by others (see Methods). We also identified the neuronal marker growth associated protein 43 (GAP43) in the lens, which has been characterized as a HuD target in neurons (Figure 7B,E-G). When we examined the distribution of HuR and HuB/C/D proteins in the lens, we again observed a predominantly complementary expression pattern for these two Hu protein groups in epithelial and fiber cells that again agrees with the distribution of other members of this interlocking regulatory network in lenses (Figure 7G). Interestingly, similar to PTB and nPTB, globally expressed HuR was also detected primarily within epithelial cell nuclei and HuB/C/D were detected in the cytoplasm along the length of peripheral fiber cells, together with GAP43 and Ca^2+^/calmodulin-dependent protein kinase IIα (CamKIIα; Figure 7G). Like GAP43, CamKIIα has also been identified as a target of HuD regulated expression in neurons [51]. In skin, Koljonen et al. [33] identified HuR predominantly in cell nuclei, and this agrees with studies that found that ectopic cytoplasmic HuR in non-neural cells is a prognostic factor in cancers [52]. For comparison, we detected HuR in skin cell nuclei (shown in Figure 5I).

### ‘Neuron-specific’ HuB/C/D and nPTB dependent alternative splicing can also occur in the lens

**Figure 8:**
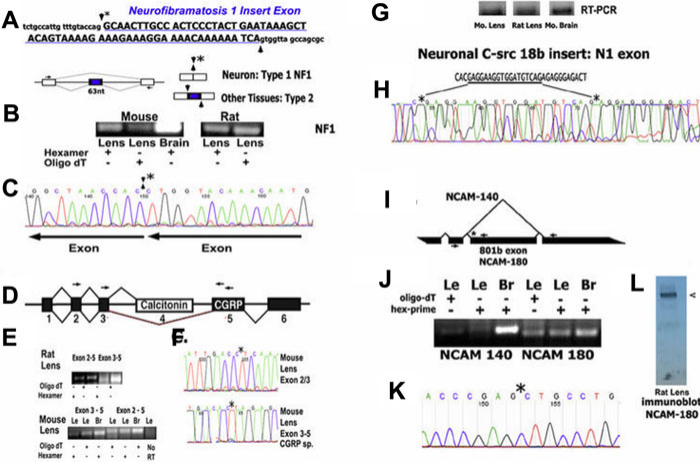
HuB/C/D and nPTB dependent alternative splicing also occurs in mouse and rat lens. **A**: Neuronal alternative splicing of *Nf1* Type 1 transcripts omits a 63 bp exon. Type 2 transcripts include this 63 bp exon, **B**: Amplification of *Nf1* transcripts from lens and brain with primers corresponding to exons adjacent to the insert exon, **C**: Sequence of amplified *Nf1* product from lens identifying Type 1 splicing, and omission of the 63 bp exon. **D**: In neurons, alternative splicing skips exon 4 to produce CGRP transcripts, **E**: Amplification of CGRP transcripts from lens and brain, **F**: Sequences identifying alternatively spliced 3–5 exon junctions in CGRP transcripts amplified from lenses. Alternative splicing of Neuronal C-src includes18 bp N1 exon. **G**: Amplification of C-src transcripts from lens and brain with primers corresponding to adjacent exons, **H**: DNA sequence of amplified Neuronal C-src product identifying the *N1* exon in the isoform produced in lenses. **I**: nPTB-dependent alternative splicing of NCAM-180 transcripts includes an 801 bp exon, **J**: Amplified NCAM-180 transcripts from lens and brain, **K**: Sequence of amplified *NCAM-180* transcripts from lens identifying the 5′ splice junction that includes the 801 bp exon, **L**: Immunoblot of NCAM-180 protein in rat lens using mAb anti-NCAM-180 specific antibody.

To begin to assess the functions of neuronal RBPs in the lens, we began by examining lenses for classic examples of alternative splicing events that have been shown to be dependent on this splicing machinery in neurons. Two examples of HuB/C/D-dependent alternative splicing reviewed by Hinman and Lou [27] involve neurofibramatosis 1 (Nf1) and the neuropeptide, calcitonin gene related peptide (CGRP). Nf1 is a negative regulator of the RAS oncogene and governs cell proliferation and differentiation. The Type 1 Nf1 alternative transcript lacking a 63b insert exon more strongly suppresses RAS activities and thus cell growth in neurons [53]. Using primers adjacent to this insert exon, our assay was capable of detecting both alternatively spliced transcripts. However, we amplified one major alternatively spliced transcript in mouse and rat lenses, similar to brain. When we sequenced this product we produced a single coherent sequence that confirmed the 63b insert exon is predominantly skipped in *Nf1* transcripts in the lens, similar to neurons (Figure 8A). We are currently examining the distribution of Type 1 *Nf1* transcripts in lenses. These findings also suggest this stronger mode of RAS down-regulation by Type 1 Nf1 may also occur in lenses for growth control. We also examined Calcitonin/CGRP (Calcitonin Gene Related Peptide) splicing reactions in lenses. CGRP is one of the most abundant neuropeptides in peripheral and central neurons [54]. In neurons, exon 4 is specifically excluded [27], and alternative splicing of exons 3 and 5 produces a transcript encoding CGRP (Figure 8,B). In lens, Rosenblatt et al. [55] also demonstrated the fiber cell specific expression of the CGRP receptor component, RCP, which co-localizes with CGRP in neurons. Using primers corresponding to exons 3 and 5, we amplified *CGRP*-specific transcripts from mouse and rat lenses, and confirmed this exon 3–5 junction sequence is also produced in the lens.

We also tested lenses for nPTB dependent alternative splicing of the Neuronal C-src oncogene and neural cell adhesion molecule (NCAM-180) [2,56-58]. Splicing reactions that include an alternative 18b N1 exon in C-src transcripts have been extensively characterized over the past 25 years as being neuron-specific. Similar to Nf1 above, we used an assay able to detect both N1^+^ and N1^-^ alternatively spliced transcripts from this region. Our experiments using total rat and mouse lens and brain RNAs produced one major amplified product that when sequenced produced a single coherent nucleotide sequence identifying the N1 exon in lens C-src transcripts (Figure 8,C). These findings provide evidence that nPTB dependent splicing reactions also occur in the lens. A second example of nPTB splicing involves the ~180 kDa isoform of NCAM (Neuronal Cell Adhesion Molecule) [2,58]. In neurons, NCAM-180 includes an 801 bp insert exon. This isoform has a key role in regulating neurite extension by working with GAP43, which we also identified in the lens [59,60]. Our examination of lenses identified the NCAM-180 transcript with correct splice junction sequences that includes the 801b exon (Figure 8,D). In addition, antibodies directed at epitopes encoded by this large exon also detected NCAM-180 protein in lenses on immunoblots.

### Increased HuD expression in transgenic mouse lenses showed increased expression of the HuD target genes GAP43 and CamKIIα in the lens

The following experiments examined the role of neuronal RBP isoforms in the lens in further detail. We examined HuD transgenic mouse lenses that exhibit increased HuD protein in their lenses. This *tg* model uses a CamKIIα promoter to drive HuD expression, and also contains a myc-epitope tag [42,61-63]. Previous studies demonstrated increased HuD expression in brain in this model led to increased protein expression of the GAP43 target gene in the hippocampus, and as a result affected spatial learning [64]. To date, CamKIIα and GAP43 have also been described as neuron-specific. For example, GAP43 is often used as a neuronal marker. We demonstrated that CamKIIα and GAP43 are produced in *wt* mouse and rat adult lenses, consistent with HuD expression in *wt* lenses (Figure 7). Both transcripts were detected in mouse and rat lenses using primers corresponding to different exons, and specific antibodies identified CamKIIα and GAP43 protein expression predominantly in fiber cells, with little in cells along the lens anterior surface. This distribution agrees with predominant expression of endogenous HuD in fiber cells. HuD also regulates protein synthesis-dependent changes required for neuronal cell elongation and plasticity [4,28,30], consistent with effects shown in brain in HuD *tg* mice. When we examined 3-month old *wt* and HuD transgenic lenses we verified increased expression of HuD, together with expected increases in GAP43 and CamKIIα in transgenic lenses. These findings are consistent with results in brain in this model (Figure 7) [63-65].

### miR-124 in vertebrate lenses

**Figure 9:**
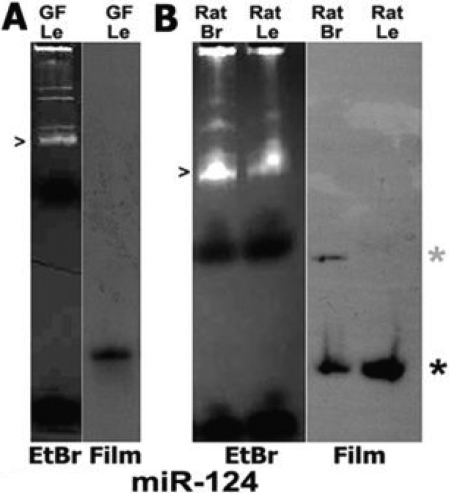
miR-124 in vertebrate lenses detected on northern blots. **A**: Left: Total RNA from *N. crassa* (goldfish) lenses resolved on acrylamide gels. Highly represented RNAs are stained with ethidium bromide indicated by arrowhead. Right: *miR-124* detected with labeled probe. **B**: Left: Ethidium bromide stained RNAs from rat lens and brain. Right: *miR-124* detected with radiolabeled *miR-124* probe. Lower asterisk: ~22 bp nucleotide *miR-124*. Upper asterisk: ~76 bp precursor in rat brain. Probes, and negative controls showing no lens expression of muscle-specific miR-1, are described elsewhere [39].

miR-124 has been studied extensively and was previously characterized as neuron-specific. miR-124 was found to interact with hundreds of non-neuronal mRNAs in neuronal cells, as well as in miR-124 transfected non-neural tissue culture cells, and suppresses their expression [36]. miR-124 can also trigger neurogenesis due to its suppression of PTB. miR-124 was shown to be further integrated into this global regulatory network in studies that demonstrated that miR-124 gene expression is also repressed by REST in non-neuronal cells [15]. In a previous study, we demonstrated miR-124 is produced in rat and mouse lenses along with other brain-enriched miRNAs, and by contrast, muscle-specific miR-1 is not present in lenses [37]. Here, we used northern blots to extend these findings and demonstrated miR-124 is produced in a wider array of vertebrate lenses (Figure 9).

## Discussion

**Figure 10:**
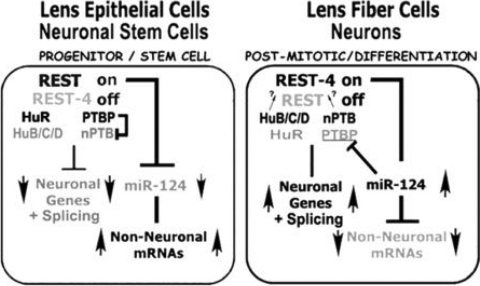
Model of regulatory interactions between factors expressed in neural progenitors and lens epithelial cells versus post-mitotic neurons and lens fiber cells. In neurons REST/NRSF transcription factors, HuR-HuB/C/D and PTB-nPTB RNA binding proteins, with miR-124 form a network that differentially regulates non-neural and neuron-specific alternative splicing and gene expression. REST suppresses >1,500 neuronal genes in non-neuronal cells throughout the body. In post-mitotic neurons REST decreases, and neuron-specific alternatively spliced REST4 is produced, further relieving repression of these genes. Ubiquitous HuR and PTB that promote non-neural splicing are also replaced by neuron-specific HuB/C/D and nPTB in post-mitotic neurons, which leads to a comprehensive reprogramming of neuron-specific alternative splicing. In non-neural cells, PTB alters nPTB transcript splicing to tag them for nonsense mediated decay. In post-mitotic neurons, REST repression of miR-124 expression is alleviated, allowing miR-124 to suppress hundreds of non-neuronal transcripts, including PTB.

We began this study with an aim toward exploring the extensive similarities in morphology that have been described in lens fiber cells and neurons, at the molecular level. We reasoned that related sets of genes and associated regulatory mechanisms may be uniquely shared in differentiating lens fiber cells and neurons to meet shared cell biology requirements for building these similar elongated cell types. To our knowledge, the present study provides the first evidence these basic regulators of neuronal gene expression that work at the transcriptional and post-transcriptional level are also expressed and used in the lens. The hallmark of this regulatory network is the restricted expression of what has been termed “neuronal” components in post-mitotic neurons, and “non-neuronal” and neural progenitor counterparts in all non-neural tissues examined to date in several diverse species (Figure 10). Studies in neurons have also shown this coordinated differential expression of REST, Hu, and PTB protein isoforms is also required for this network to function, and the present findings provide evidence this coordinated distribution of regulatory factors is matched in lens epithelial cells and post-mitotic fiber cells. Consistent with functions of these factors in neurons, we found that REST-regulated genes are also expressed in the adult lens, and largely restricted to fiber cells where REST is down-regulated. We also provided evidence that RNA splicing reactions that are determined by “neuronal” RBP isoforms can also occur in the lens. Moreover, our experiments that could simultaneously detect “neural” and “non-neuronal” alternative C-src and Nf1 transcripts showed that Neuronal C-src and Type 1 Nf1 also predominate in the lens. In neurons, the down-regulation of REST, PTB, and HuR as cells exit the cell cycle, which is followed by increased production of REST4, nPTB and HuB/C/D in post-mitotic neurons has been specifically linked with the regulation of neurogenesis.

When we considered how this REST/RBP/miRNA network might be differentially regulated in lens and brain, we speculate a possible mechanism may involve use of different canonical Wnt proteins in lens and brain. The present study determined that REST is predominantly detected in anterior lens epithelial cells and decreases at the lens anterior/posterior equator. Studies have shown REST expression can be activated by canonical Wnt signaling in neurons [66]. Canonical Wnt proteins stabilize β-catenins and affect chromatin organization and gene activation in the nucleus. Consistent with this, a DNA element identified in the REST gene locus mediates responses to Wnt/β-catenin actions [66,67]. In studies in the lens, Fokina and Frolova [68] showed canonical Wnt-2 is expressed in anterior epithelial cells in the chick, and de Iongh and coworkers [69] determined β-catenin is localized to the same cells in the mouse lens. In addition, Cyclin D1 also demonstrates similar Wnt regulation in lenses, where Wnt/β-catenin was also found to regulate Cyclin D1 [66], and like REST is expressed in anterior lens epithelial cells [70]. Conversely, when considering mechanisms that can antagonize canonical Wnt/β-catenins, and account for decreased REST at the lens equator, Fokina and Frolova [68] also determined that non-canonical Wnt-5 expression is confined to surface epithelial cells at the lens equator. It has been shown that Wnt-5 can antagonize canonical Wnt/β-catenin action [71-73]. Thus, we hypothesize that antagonistic effects of canonical Wnt and β-catenin proteins and non-canonical Wnt-5 proteins near the lens equator may work to limit REST expression in progenitor epithelial cells, similar to Cyclin D1. This mode of regulation could also account for differential control of REST in lens and brain. In contrast to Wnt-2 in lenses, neural tissues express canonical Wnt-1 and Wnt-3 that have been closely linked with neuronal development in the brain [68,74]. Thus, regulation of lens *vs*. brain canonical Wnt factors may differentiate REST activities in these two organs.

Although mechanisms that can account for expression and alternative splicing of REST in progenitor cells and REST4 in post-mitotic cells have not yet been determined, studies that generally examined interactions between the transcriptional and post-transcriptional machinery have shown that transcription and alternative splicing can be closely linked both temporally and spatially in the nucleus. These studies indicated further that cell type- or cell cycle-specific promoter occupation by different transcription factors leading to different chromatin structures, have a key role in determining alternative splicing, in addition to transcription [75]. Future studies can determine if these processes also contribute to switching REST/REST4 splicing during lens fiber cell differentiation, and what role different Wnt and catenin factors might contribute to these processes.

Our findings of preferential Type 1 Nf1 and Neuronal C-src splicing in lenses may also indicate related roles in the lens to regulate cell growth. Type 1 Nf1 inhibits RAS to a greater extent than type 2 Nf1 that includes this insert exon [53], and evidence Nf1 has a role in lens growth and differentiation was reported by Carbe (B126; Soc. for Dev. Biol. Annual Meeting, Phil. PA, 2008) who showed Nf1 is required for early murine lens development. Similarly, Neuronal C-src interactions with Synapsin 1 in neurons regulates membrane vesicle functions [76], and nPTB-dependent splicing of the N1 18b exon into a Csrc SH3 binding domain inhibits these interactions in neurons. In light of Neuronal C-src and syn1 we identified in lenses, these proteins may have similar regulatory interactions in this additional tissue.

The preferential cytoplasmic distribution of HuD protein in lens fiber cells is also consistent with HuB/C/D distribution in neuronal processes. HuD co-localizes with GAP43 transcripts in neuronal cell bodies [77,78], and is required for neuronal differentiation. These observations are also consistent with increased GAP43 protein in the hippocampus of HuD *tg* mice [79]. Our further experiments to test for HuD functions in lenses showed that increased HuD expression in *tg* lenses produced predicted increased expression of known GAP43 and CamKIIα targets in the lens, again agreeing with HuD effects in the brains of these mice. Likewise, localization of PTB in cell nuclei of non-neuronal cells [80], and cytoplasmic localization of nPTB in neurons [81] also agrees with the present data that identified PTB predominantly in lens epithelial cell nuclei, similar to their nuclear localization in skin. In contrast, we did not detect REST protein primarily in lens epithelial cell nuclei. When we investigated this further by examining similar REST expression in cells in the cornea and skin, we found the same distribution in these cell types: REST was also present throughout these cells without specific localization in cell nuclei. Conversely, these findings were matched by our demonstration of nuclear localization of PTB and HuR in lens epithelial cells, cornea and skin. Classically, transcription factors are thought to act when they are in direct contact with DNA in the nucleus, in a manner analogous to the lac operon repressor. However, considering the role of the REST/NRSF-SWI/SNF chromatin remodeling complex in organizing chromatin to affect REST gene repression [82,83], REST repression appears not to follow this classic model. Mandel and coworkers demonstrated that in some cells REST is degraded to levels just sufficient to maintain target gene chromatin in an inactive state, but still poised for expression. However, we note the present study appears to be the first to examine REST subcellular localization in lens, skin and cornea in vivo.

The present findings also raise questions about the relationship between lens cells and neurons and the evolutionary origins of these regulatory genetic switches that have been extensively characterized as having a fundamental role in establishing and distinguishing neuronal cell identity. ‘Camera eyes’ that contain cellular lenses in front of a photoreceptor cell layer have remarkably similar construction in animals as diverse as jellyfish, cephalopods and mammals [9,10,84]. In reviews on the topic of eye evolution, Gehring [84] proposed that sensory organs including the eye may have preceded the evolution of a brain. In light of these observations, the present findings may indicate the fundamental transcriptional and post-transcriptional regulatory factors examined here may also have had evolutionary origins in the lens. Future studies will be required to characterize the extent of shared gene expression and alternatively spliced products regulated by these neuron/lens-specific regulatory factors that are uniquely expressed by lens cells and neurons. This will allow the identification of functional groupings of these gene products, which in turn can inform us further about how this unique relationship between lens cells and neurons developed regarding the evolution and/or acquisition of these genetic switch components in these two cell types.

Each of these genetic switch mechanisms examined here has also been linked with neurodegenerative and neuropsychiatric conditions [27,85-87]. For example, REST and its functions have been linked with Huntington’s disease, stroke, Down syndrome, and seizures. The similarities between lens fiber cell and neuronal cell morphology, together with this unexpected level of shared neuronal gene expression and basic regulatory factors, suggest lens cells and neurons produce corresponding phenotypes resulting from genetic lesions linked with specific members of this regulatory network, and can also undergo related disease processes in response to systemic stress, disease, and aging.

## Appendix 1. Primer sequences for TR-PCR.

To access the data, click or select the words “Appendix 1.” This will initiate the download of a Microsoft Word (.doc) file that contains the data.
